# Thyroid-like Follicular Carcinoma of the Kidney: The Follicles Are out There in the Kidney—Now What?

**DOI:** 10.3390/diagnostics15091111

**Published:** 2025-04-27

**Authors:** Stefan Spiric, Bojana Rancic, Branko Kosevic, Ivica Nikolic, Snezana Cerovic, Bozidar Kovacevic

**Affiliations:** 1 Institute of Pathology and Forensic Medicine, Military Medical Academy, 11000 Belgrade, Serbia; stfnspiric@gmail.com (S.S.); bojana.jovanovic.vma@gmail.com (B.R.); cerovics@gmail.com (S.C.); 2 Medical Faculty of the Military Medical Academy, University of Defence, 11000 Belgrade, Serbia; bkosevic@gmail.com; 3 Clinic of Urology, Military Medical Academy, 11000 Belgrade, Serbia; inikolic1970@gmail.com

**Keywords:** renal cell carcinoma, thyroid-like follicular carcinoma, differential diagnosis, immunohistochemistry

## Abstract

Thyroid-like follicular carcinoma of the kidney (TLFC-K) is a rare primary kidney carcinoma with fewer than 60 reported cases. Current data suggest that TLFC-K has low malignant potential, with only a few reported cases of unfavorable clinical behavior. Histologically, TLFC-K is indistinguishable from kidney metastasis of well-differentiated follicular cell-derived thyroid carcinomas. Furthermore, folliculo-tubular patterns can be seen in different types of kidney lesions, making assessing follicular architecture in the kidney diagnostically challenging. We present a case of TLFC-K with a list of differential diagnoses. A hyperechoic tumor was found incidentally in the upper pole of the right kidney of a 66-year-old man. The patient underwent a radical nephrectomy. Histologically, the tumor was well-circumscribed, composed of follicular/tubular structures of different sizes filled with colloid-like material. Immunohistochemically, the absence of a positive reaction for thyroglobulin and TTF-1 excluded the secondary origin of the tumor from the thyroid. Tumor cells also showed diffuse positivity for vimentin and PAX8 and focal positivity for CK7 and CD10. The results of all other applied immunostaining tests did not align with those of different types of kidney tumors that may exhibit predominantly follicular patterns. Accordingly, TLFC-K was diagnosed. The patient shows no signs of disease relapse at the 5-month follow-up.

**Figure 1:**
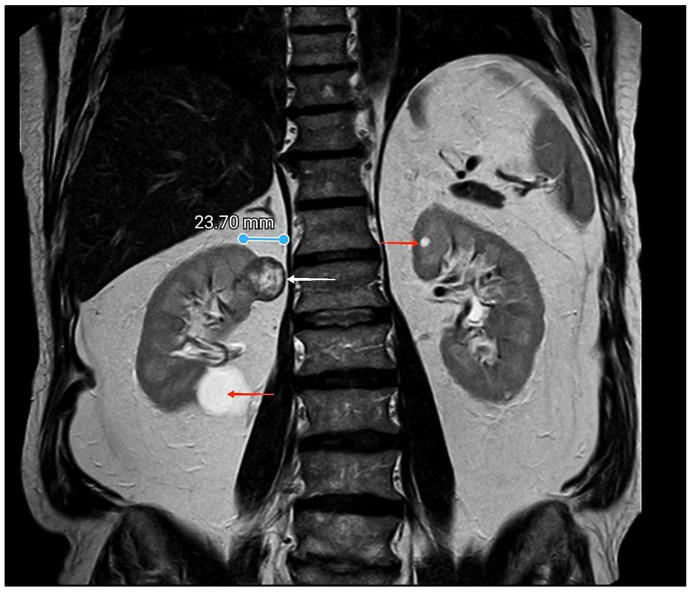
Magnetic resonance imaging (MRI) of the abdomen. A 66-year-old man presented to his urologist for a regular benign prostate hyperplasia work-up. A well-circumscribed and dominantly hyperechoic mass lesion with an approximate diameter of 25 mm was discovered incidentally in the upper pole of the right kidney on an abdominal ultrasound. The MRI scan verified the well-demarcated and solid lesion measuring 27 mm × 22 mm with increased signal intensity on T2-weighted sequences with respect to the kidney parenchyma (white arrow). A simple cyst with a diameter of 32 mm was described in the lower pole of the same and opposite kidney (red arrows). The complete blood count, comprehensive metabolic panel, and urinalysis were unremarkable. The patient had no malignancies in their past medical history. Partial nephrectomy was indicated. Intraoperatively, extensive adhesions to the peritoneum and the liver were identified. Due to the patient’s habitus, the superior pole of the kidney was in a more subhepatic position than presented by preoperative imaging. Also, adherent perinephric fat was determined. The presence of inseparable adipose tissues adhered to the kidney parenchyma, combined with the tumor location at the posterior side of the upper pole, made it impossible to provide an adequate plane to perform a partial nephrectomy. Taking that into consideration and to also decrease the possibility of excessive intraoperative bleeding, the decision was made to proceed with radical nephrectomy. The patient underwent radical nephrectomy without complications in the postoperative course.

**Figure 2:**
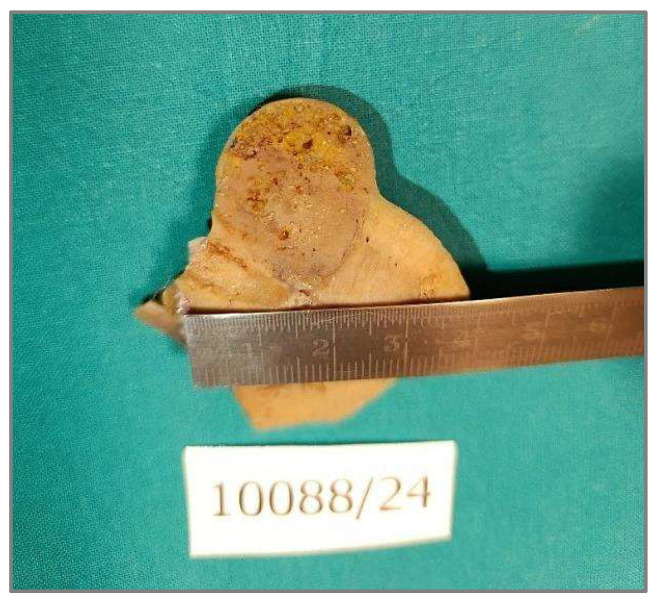
Grossly, the tumor was well-circumscribed, measuring 28 mm in greatest dimension, tan-yellow, with a microcystic and colloidal appearance on the cut surface.

**Figure 3:**
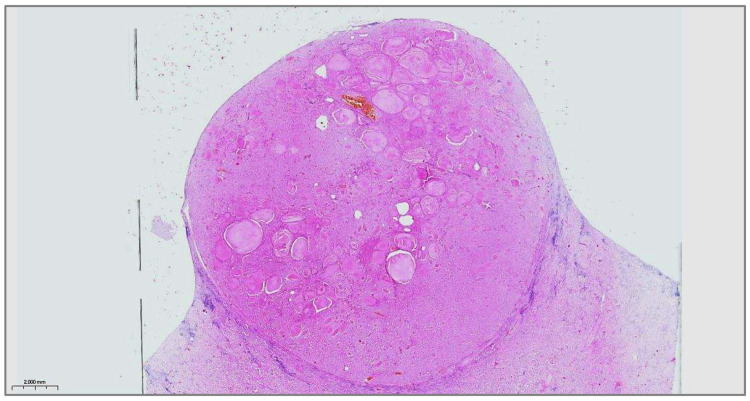
Microscopic scan of the whole tumor section surface at a magnification of ×20, H&E*. A thin, fibrous capsule encircles the tumor entirely made up of follicles of various sizes, occasionally displaying a microcystic appearance. Intraluminally, colloid-like material is noticeable in the microcystic and follicular spaces. The microscopic appearance of the tumor is indistinguishable from a thyroid follicular nodule. However, its localization within the kidney parenchyma indicated kidney metastasis of well-differentiated follicular cell-derived thyroid carcinoma (WD-FCDTC). Kidney metastases of all types of WD-FCDTC have been reported as an exceedingly rare phenomenon. Papillary thyroid carcinoma (PTC), the most common type of WD-FCDTC, is more frequently reported than oncocytic and follicular thyroid carcinoma and typically occurs in patients with already disseminated metastatic disease [1,2,3]. PTC usually metastasizes via lymphatics into the neck lymph nodes and, less frequently, hematogenously into the lungs and bones [4]. Solitary renal metastases of PTC are unexpected findings, with only a limited number of reports [5]. Nevertheless, given that PTC is generally biologically indolent and has an excellent prognosis with a 20-year survival rate of >90%, the occurrence of renal tumors in PTC survivors should raise suspicion of late PTC metastasis into the kidney as a differential diagnosis for a new primary renal neoplasm [4,5,6]. In cases where kidney metastasis of PTC or other types of WD-FCDTC is preoperatively confirmed, standard therapy includes radioactive iodine therapy. Yet, in a small subset of PTC patients with a history of low iodine avidity WD-FCDTC, surgery for renal metastasis may be considered as an option [2,3,5]. * Hematoxylin and eosin stain.

**Figure 4:**
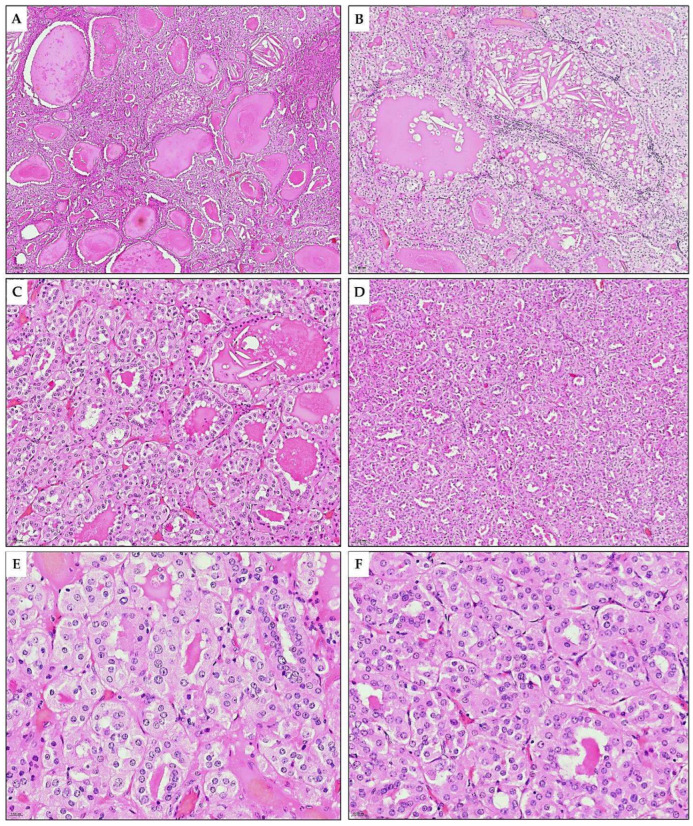
Microscopic appearance of TLFC-K. (**A**) Parts of the tumor exhibit predominantly macrofollicular and microcystic patterns. The follicular/microcystic spaces were filled with eosinophilic, amorphous, colloid-like material (H&E*, ×5). (**B**) Few microcystic spaces contain cholesterol clefts and foamy macrophages. Perifollicularly, tumor-infiltrating lymphocytes are present (H&E, ×10). (**C**) Medium-sized follicles are overlaid with eosinophilic cuboidal and low-columnar cells (H&E, ×20). (**D**) The tumor area is composed of small follicles with the same cytological features (H&E, ×10). (**E**,**F**) The oval or polygonal cells lining the tubular structures were medium-sized to large, with abundant cytoplasm of varying degrees of eosinophilia. Nuclei are oval with evenly distributed chromatin and visible nucleoli (H&E, ×40). * Hematoxylin and eosin stain.

**Figure 5:**
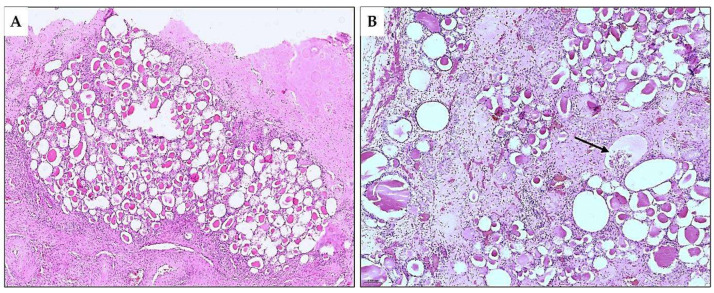
Differential diagnosis of TLFC-K. In addition to kidney metastasis of well-differentiated follicular cell-derived thyroid carcinomas, the differential diagnosis of TLFC-K includes various benign and malignant primary renal lesions [7]. Kidney thyroidization is the earliest described pattern of atrophic kidney that mimics thyroid tissue [8]. The thyroidization can sometimes be extensive and atrophic collecting ducts filled with eosinophilic content may form nodular areas, as shown in (**A**) (H&E*, ×5). When thyroidization shows a nodular arrangement, the differential diagnosis includes TLFC-K and a rare morphological entity designated as an atrophic kidney-like lesion (AKLL), currently considered a benign renal lesion with indolent behavior [9]. Differentiation of thyroidization from both TLFC-K and AKLL is microscopically aided by its diffuse appearance, absence of a capsule, as well as the detection of glomeruli (arrow) and altered vascular spaces in the interstitium as shown in (**B**) (H&E, ×10). Some cases of AKLL were previously classified as TLFC-K [10,11,12]. In differentiating these two entities, the differences in tumor cells are significant. In AKLL, the cells are flattened or low-cuboidal, while the cells of TLFC-K are larger, polygonal, or cylindrical. Moreover, AKLL can be distinguishable by entrapped atrophic benign tubules and by the positive immunohistochemical WT-1 staining [9,12]. * Hematoxylin and eosin stain.

**Figure 6:**
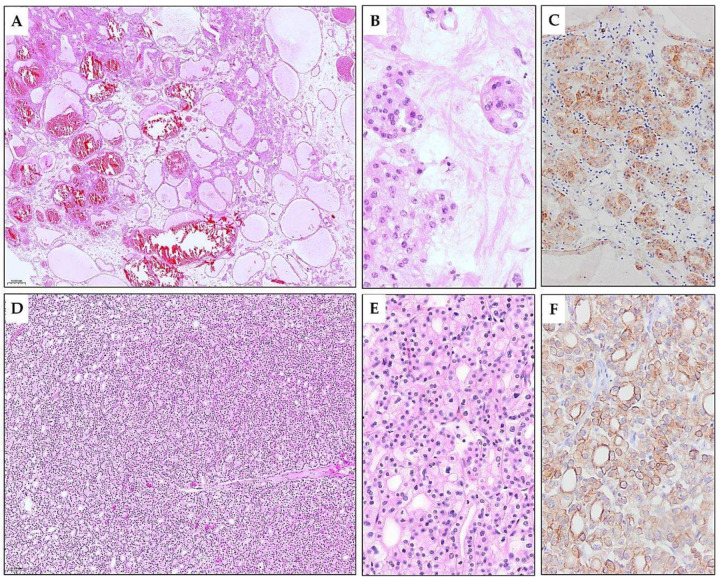
Differential diagnosis of TLFC-K. (**A**) Renal oncocytoma displaying dilated tubules and microcystic pattern. Tubular spaces were filled with pale eosinophilic fluid or blood (H&E*, ×10). This morphological pattern of renal oncocytoma is rare, and in the differential diagnosis, it should include various types of renal cell carcinoma with tubulocystic patterns, including TLFC-K [13]. The characteristic presence of typically small solid nests in a loose hypocellular and hyalinized stroma (**B**) (H&E, ×40) and specific immunophenotype that included immunopositive stain for CD117 supports the diagnosis of oncocytoma (**C**) (H&E, ×20) [7]. (**D**) Chromophobe renal cell carcinoma is a primary renal carcinoma with many morphological faces [14]. One of its architectural variants may predominantly be composed of tubular and follicular-like spaces when it mimics a TLFC-K (H&E, ×10). (**E**) Searching for typical cellular features, such as prominent cell membranes and raisinoid nuclei surrounded by perinuclear haloes, may aid in the diagnosis (H&E, ×40) [7]. (**F**) Immunopositivity for CK7 (shown in figure) and CD117 can lead to a correct diagnosis of chromophobe carcinoma. * Hematoxylin and eosin stain.

**Figure 7:**
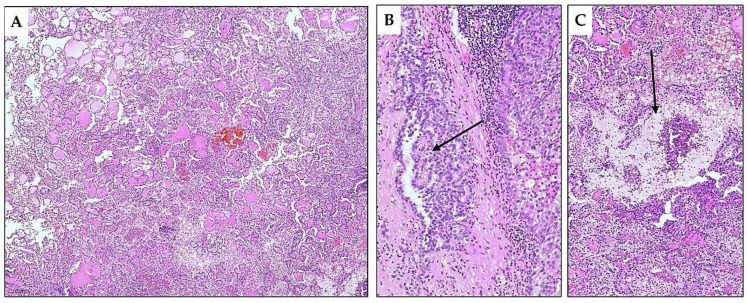
Differential diagnosis of TLFC-K. (**A**) Cross-sections of papillary structures with variable degrees of edematous fluid collection within the cores of papillae sometimes dominate in papillary renal cell carcinoma, mimicking thyroid histology and TLFC-K. This papillary renal cell carcinoma pattern is the most commonly reported pitfall in the diagnosis of TLFC-K, causing concerns that at least some of the previously reported TLFC-K are papillary renal cell carcinoma [15,16,17,18]. (**B**) Paying attention to the tumor parts displaying well-forming papillary structure (arrow) (H&E*, ×20) and (**C**) detection of stromal foamy histiocyte collections (arrow) can help practicing pathologists to avoid the risk of misdiagnosis (H&E, ×10). From a practical perspective, diagnosing renal tumors with uncommon morphology typically necessitates additional tissue processing and a thorough search for recognizable microscopic tumor characteristics to achieve an accurate diagnosis. Precise diagnostics determine prognosis and treatment options for kidney tumors. Table S1 summarizes the histological features of kidney tumors and tumor-like lesions, which may exhibit focal or diffuse tubular or follicular morphology, potentially mimicking thyroid tumors and demonstrating varying prognoses across different tumor entities [7,12,19,20,21,22,23].

**Figure 8:**
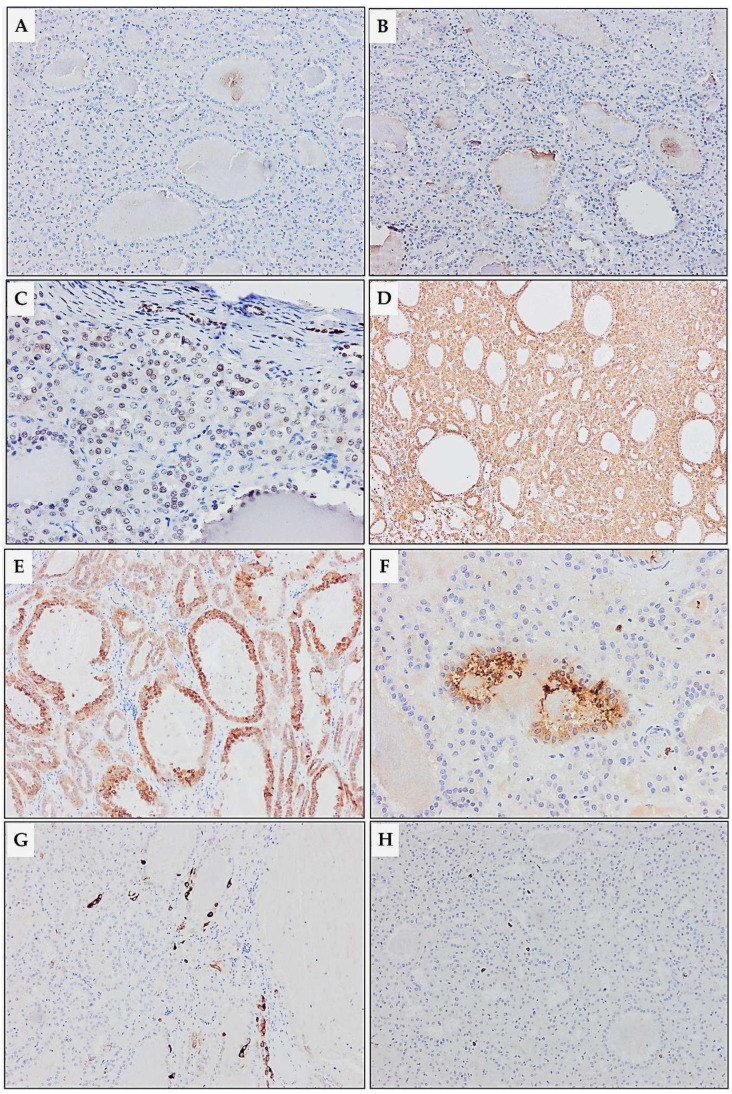
Immunohistochemical analysis of TLFC-K. (**A**) Thyroglobulin (×4), (**B**) TTF-1 (×10), (**C**) PAX8 (×20), (**D**) Vimentin (×4), (**E**) AMACR (×10), (**F**) CD10 (×10), (**G**) CK7 (×20), and (**H**) CD117 (×10). Immunohistochemically, the lack of positivity for thyroglobulin and TTF-1 excluded the tumor’s metastatic origin from the thyroid. Additionally, in the present case, the kidney tumor was a solitary lesion, lacking the characteristic nuclear and architectural features of PTC. This is the opposite of most of the reported cases of metastatic thyroid carcinomas in the kidney that presented as disseminated metastatic disease and were of the papillary type [1,2]. The tumor showed weak to moderate nuclear positivity for PAX8 and diffuse strong positivity for vimentin, aligning with previously reported cases [12,22,23]. Positivity for AMACR was also observed, contrary to most previous reports [7,12,22,23]. Although positivity for AMACR is characteristic of papillary renal cell carcinoma, it can also be observed in various renal tumors, including 17% of TLFC-K, suggesting its nonspecificity in differential diagnostics [12,23,24,25]. In the present case, focal positivity for CK7 and CD10 aligns with the heterogeneity of TLFC-K. Specifically, positivity for CK7 and CD10 in TLFC-K is reported differently in the literature, ranging from nonreactive to diffusely positive; therefore, they cannot be regarded as specific markers for diagnosing TLFC-K [12,20,22,23]. Additionally, we did not detect loss of immunopositivity of tumor cells for succinate-dehydrogenase B and fumarate-hydratase; tumor cells were negative for WT1, CD56, RCC, and CK19. The immunohistochemical characteristics of TLFC-K are not specific and should be interpreted in the context of tumor morphology. Table S2 details the immunohistochemical findings for renal tum.ors with tubular/follicular patterns [7,12,22,23]. For twenty years now, TLFC-K has remained an emerging entity not fully clinicopathologically portrayed [7,20]. Thanks to our current knowledge, it is mainly an incidentally discovered indolent tumor confined to the kidney [12,23]. Yet, few reported cases have been disclosed as locally advanced diseases with local and/or distant metastasis [21,26,27,28,29]. One must report future cases in order to complete morphological criteria and immunohistochemical findings for precise diagnosis of TLFC-K. Our understanding of the TLFC-K genetic landscape is still rather rudimentary. Al-Obaidy et al. carried out the most extensive molecular testing to date [30]. They analyzed three TLFC-K cases by next-generation sequencing and/or reverse-transcription PCR and showed in all of them a recurrent EWSR1-PATZ1 gene fusion for the first time. Finding this intrachromosomal rearrangement is an encouraging milestone. On the whole, more studies on morphology, immunohistochemistry, and molecular characterization with long-term follow-up are needed to enlighten our curiosity about TLFC-K.

## Data Availability

Data are contained within the article or supplementary material.

## Supplementary Materials

The following supporting information can be downloaded at: https://www.mdpi.com/article/10.3390/diagnostics15091111/s1, Table S1: Most characteristic histological features and biological behavior of kidney tumors and tumor-like lesions that may mimic thyroid tumors; Table S2: Immunohistochemical findings for renal tumors and tumor-like lesions that may mimic thyroid tumors.
