# Supplementary material for: Thyroid-like Follicular Carcinoma of the Kidney: The Follicles Are out There in the Kidney—Now What?
Source: Diagnostics (Basel). 2025 Apr 27;15(9):1111. doi: 10.3390/diagnostics15091111 (PMC12071713; doi:10.3390/diagnostics15091111)
Supplement: Supplementary file 1 [file diagnostics-15-01111-s001.zip › diagnostics-3576158-supplementary.pdf]

Table S1. Most characteristic histological features and biological behavior of kidney tumors and tumor-like lesions that may mimic thyroid tumors

Lesion type	Predominant histologic pattern	Cytologic features	Stromal features	Clinical behavior
TLFC-K	Follicular/tubular	Cuboidal or low columnar cells; moderate amount of amphophilic-to-eosinophilic cytoplasm	Inconspicuous	Mostly indolent; rarely metastatic
PRCC	Papillary, >50% variable proportion of tubular and/or solid growth pattern	Small cuboidal cells with lightly basophilic cytoplasm or large, eosinophilic cells with pseudostratification	Collections of foamy macrophages, psammoma bodies and hemosiderin pigment	Malignant; more favourable than CCRCC
Oncocytoma	Solid nests/alveolar; commonly focally microcystic or tubular	Eosinophilic, granular cytoplasm; round, uniform nuclei with vesicular chromatin; Central nucleoli are often present	Stroma hypocellular, often hyalinized	Benign
ChRCC	Solid nests/alveolar	Cells with pale, finely reticular or densely eosinophilic, granular cytoplasm; raisinoid nuclei with perinuclear halos; prominent cell membranes;	Thin, incomplete fibrovascular septa	Malignant, more favourable than CCRCC and PRCC
EoCRCC	Solid nests/alveolar, focally tubules, micro- or macrocysts	Clear to granular/eosinophilic cytoplasm, hyaline globules	Intricate, branching fibrovascular septations	Malignant, depending on stage and grade
Tubulocystic RCC	Small- to intermediate-sized, cystically dilated tubules	Single layer of flat, hobnail, or cuboidal to columnar cells	Stroma hypocellular, usually fibrotic	Mostly indolent; rarely metastatic
FH-dRCC	Papillary, tubulocystic, tubulopapillary, solid and cribriform	Large nuclei with prominent nucleoli surrounded by a clear perinucleolar halo (CMV-like inclusion)	Papillary cores hyalinized; no foamy macrophages.	Malignant; mostly aggressive
SDH-dRCC	Nested or solid sheet-like	Eosinophilic cells with clear (flocculent) cytoplasmic inclusions; Round nuclei	Stromal mast cells; rarely mucin.	Mostly indolent; rarely metastatic
AKLL	Macro- and microfolllicular; Atrophic kidney appearance	Flat and atrophic cells; Commonly with floating, intraluminal cells	Dense collagenous stroma with atrophic tubules and/or collapsed glomeruli	Benign

RCC – Renal cell carcinoma; TLFC-K – Thyroid-like follicular carcinoma of the kidney; PRCC – Papillary RCC; CCRCC – Clear cell RCC; ChRCC – Chromophobe RCC; EoCRCC – Eosinophilic variant of clear cell RCC; FH-dRCC – Fumarate hydratase-deficient RCC; SDH-dRCC – Succinate dehydrogenase-deficient renal cell carcinoma; AKLL – Atrophic kidney-like lesion; N/A – Not applicable

Table S2. Immunohistochemical findings for renal tumors and tumor-like lesions that may mimic thyroid tumors

	**TLFC-K**	**PRCC**	**Oncocytoma**	**ChRCC**	**EoCRCC**	**Tubulocystic RCC**	**FH-dRCC**	**SDH-dRCC**	**AKLL**
PAX8	+	+	+	+	+	+	+	+	−
CK7	+	+	−/scattered cell +	+	−/may be focally + in cystic areas	+	−	−	−
CD10	−	+	+	−/+	+	+	+	−	N/A
AMACR	−	+	−	−	−/may be focally +	+	+	−	N/A
CAIX	N/A	−	−	−	+	N/A	−	−	N/A
Vimentin	+	+	−	−	+	+	variable	−	N/A
CD117	-	−	+	+	−	−	−	−	N/A
WT1	−	−	N/A	N/A	N/A	N/A	−	N/A	+
RCC	−	+	N/A	−	+	N/A	N/A	N/A	N/A
FH	+	+	+	+	+	+	−	+	+
SDHB	+	+	+	+	+	+	+	−	+

RCC – Renal cell carcinoma; TLFC-K – Thyroid-like follicular carcinoma of the kidney; PRCC – Papillary RCC; CCRCC – Clear cell RCC; ChRCC – Chromophobe RCC; EoCRCC – Eosinophilic variant of clear cell RCC; FH-dRCC – Fumarate hydratase-deficient RCC; SDH-dRCC – Succinate dehydrogenase-deficient renal cell carcinoma; AKLL – Atrophic kidney-like lesion; N/A – Not applicable
